# Development of a targeted HPLC-ESI-QqQ-MS/MS method for the quantification of sulfolipids from a cyanobacterium, selected leafy vegetables, and a microalgae species

**DOI:** 10.1007/s00216-021-03164-3

**Published:** 2021-01-22

**Authors:** Judith Fischer, Mascha Treblin, Tobias Sitz, Sascha Rohn

**Affiliations:** 1 Hamburg School of Food Science, Institute of Food Chemistry, University of Hamburg, Grindelallee 117, 20146 Hamburg, Germany; 2 Institute for Food and Environmental Research (ILU) e.V., Papendorfer Weg 3, 14806 Bad Belzig, Germany; 3 Department of Food Chemistry and Analysis, Institute of Food Technology and Food Chemistry, Technische Universität Berlin, Gustav-Meyer-Allee 25, 13355 Berlin, Germany

**Keywords:** Cyanobacteria lipids, Sulfoquinovosyldiacylglycerols, HPLC-ESI-QqQ-MS/MS, Method validation

## Abstract

The use of macro- and microalgae, as well as cyanobacteria, becomes increasingly important for human nutrition, even in Western diets. Health effects, positive as well as negative, are believed to result mainly from minor components in the food. In macro- and microalgae as well as in certain cyanobacteria, one class of such minor compounds is sulfolipids, more precisely sulfoquinovosylmonoacylglycerol (SQMG) and sulfoquinovosyldiacylglycerol (SQDG) derivatives. SQMGs and SQDGs consist of a diacylglycerol esterified with varying fatty acid combinations and a sulfoquinovose moiety. Sulfoquinovose is a sulfonated hexose analogous to d-glucose, but featuring a stable carbon-sulfur bond. With regard to their chemical structure, SQDGs can be distinguished according to their sn1- and sn2-bound fatty acids. Although there is great interest in SQDGs, because of their controversially discussed bioactivities, only a negligible number of comprehensive methods for identification and quantification has been published, so far. Within this work, a sample preparation including a quantitative isolation of SQDGs from selected raw materials, a clean-up with solid-phase extraction (SPE), and a sensitive liquid chromatography-tandem mass spectrometry (LC-MS/MS) method for simultaneous identification and quantitation of different, intact SQMGs and SQDGs were developed and validated. The applicability of the method was further demonstrated by comparing a prominent cyanobacterium (*Arthrospira* sp.) with a microalgae preparation (*Chlorella vulgaris*), and selected leafy vegetables (spinach, basil).

## Introduction

As early as in 1974, *Arthrospira* sp. (filamentous cyanobacteria) was described by the *United Nations World Food Conference* as possibly the best food for the future. The use of microalgae and cyanobacteria for human nutrition has a traditional past in several countries and offers many possibilities and advantages for human nutrition. In Western diets, they are mostly being consumed for their functional benefits beyond traditional considerations of nutrition and health [1]. In Europe, only some algal and cyanobacterial species are legally on the market as food ingredients. However, some products have been already legalized according to the Regulation (EU) 2015/2283 of the *European Commission* as novel food such as oil from the microalgaes *Ulkenia* sp. or *Odontella aurita* [2]. The positive effects on human health are hypothesized to result from certain macronutrients, but moreover, from their micronutrients. However, the latter are somehow controversially discussed in the literature. There is often little known about negative aspects or minor compounds with adverse properties, although they are significantly metabolized in the human organism [3]. Sulfoquinovosyldiacylglycerol derivatives (SQDGs) might represent one of those minor compounds with possible bipolar properties in algae products. They consist of sulfoquinovose and a diacylglycerol, whereas sulfoquinovose is a sulfonated hexose analogous to d-glucose with a stable carbon–sulfur bond. The meaning of sulfolipids for the human diet is still controversially discussed. On the one hand, they are attributed to some health-promoting effects and are known to have antiviral properties [4]. On the other hand, sulfur compounds can have a negative impact, because some microorganisms of the lower gastrointestinal tract can produce hydrogen sulfide as a (toxic) metabolite during the biotransformation in the gut [5]. Therefore, knowledge about the impact of SQDGs on human health might still be a crucial factor. To evaluate potential physiological effects, it is essential to obtain reference compounds in comparatively high amounts and appropriate purity and to establish methods enabling a reliable quantification of SQDGs.

The analytical approaches on SQDGs began in 1959 with the discovery of novel sulfolipids by Benson et al. They described SQDGs for the first time and cultivated photosynthetic microorganisms and higher plants in the presence of isotopically ^35^S-labeled sulfate. A lipid extract was separated with paper chromatography and they investigated the chemical composition and molecular structure using various radiochromatographic methods [6]. In the late 1960s, many chromatographic and crystallographic studies followed to learn more about the structure and function of the newly described sulfolipids [7, 8]. In 1989, the *US National Cancer Institute* in Bethesda, MA, classified sulfolipids as potential antiviral agents [4]. Many studies on possible pharmacological relevance were conducted accordingly [9–11]. Generally, an isolation procedure was used to obtain SQDGs as lipid extracts, but more detailed investigation of the individual lipid compounds was not carried out [12]. However, when sulfolipids were quantified, it was only done as sum parameter with methods that did not differentiate between the single SQDG. Both thin-layer chromatographic methods and liquid chromatography were used [13–17]. In addition to the quantification as a sum parameter, some publications describe identification of selected fatty acids using gas chromatography [18, 19]. GC analysis of (intact) sulfolipids, apart from the analysis of their fatty acids only, has not been done so far, because molecular structure disables evaporation. However, investigations of similar compounds like monogalactosyldiacylglycerols (MGs) and digalactosyldiacylglycerols (DGs) using GC-MS have been performed and published. With the help of GC-MS methods, it was also possible to detect and quantify galactolipids, even though they require extensive pre-analytical procedures such as purification and derivatization for yielding volatile analytes [19, 20]. Collision-induced dissociation tandem mass spectrometry (CID-MS/MS) was used to further investigate sulfolipids from complex lipid extracts. It provided structural information about the fatty acids in sn1 and sn2 positions. During fragmentation by CID-MS/MS, similar fragments (*m/z* 225, 165, 153, 95, and 81), being formed from the sulfolipids, were observed. These fragments result from the fragmentation of the sulfoquinovose group and are therefore specific for all sulfolipids. Furthermore, fragments attributed to the neutral loss of fatty acids were observed, as well. These allow conclusions to be drawn about the fatty acid composition of the corresponding sulfolipids [21–23].

It was further determined by CID-MS/MS that the fragment resulting from the fragmentation of the sn1 fatty acid shows a higher intensity, and thus, the regiochemistry of the glycerol backbone of the SQDG can be inferred [21]. Recent studies have focused on the identification of new SQMGs and SQDGs using lipodomic approaches [24–26]. Nonetheless, there is actually no method for quantifying multiple intact SQMGs and SQDGs for evaluating phytochemical or food chemical questions.

Consequently, the aim of this study was to develop an accurate and sensitive quantification method for the measurement of different intact SQDGs by liquid chromatography coupled to mass spectrometry, following the extraction from cyanobacteria and microalgae. Additionally, selected plant materials were analyzed. These are believed to synthesize also sulfolipids to a certain extent, being closely related to chlorophyll synthesis. The different matrices are compared for getting an impression about distribution, and also for evaluating, if formation of SQMGs and SQDGs is differing, depending on source, specific lipid metabolism, and accompanying substances/substrates.

## Materials and methods

### Chemicals

Acetonitrile, chloroform, methanol, acetic acid, phosphoric acid, and hydrochloric acid were purchased from Carl Roth GmbH & Co KG (Karlsruhe, Germany). Ammonium acetate, copper sulfate, and potassium chloride were purchased from Sigma-Aldrich GmbH (Munich, Germany). The standard substances SQDG 816 (2-*O*-hexadecanoyl-1-*O*-(9Z,12Z,15Z-octadecatrienoyl)glycerol 3-(6-deoxy-6-sulfo-α-d-glucopyranoside)) and 3-*O*-sulfo-d-galactosyl-β1-1′-N-heptadecanoyl-d-erythro-sphingosine as internal standard (ISD) were purchased from Avanti Polar Lipids Inc. (Alabaster, AL, USA). All aqueous solutions were prepared with deionized water, generated by a Purelab flex water purification system (Veolia Water Technologies Deutschland GmbH, Celle, Germany). NH_2_ cartridges (6 mL, 500 mg) were purchased from Macherey-Nagel GmbH & Co. KG (Düren, Germany).

### Preparation of standard and calibration solutions

Primary standard stock solutions were prepared by dissolving 5 mg of the commercially purchased standard SQDG 816 in 5 mL acetonitrile, resulting in a final concentration of 5 mg/mL (5000 ppm). The calibration standard stock solution was diluted with acetonitrile to achieve equidistant concentration in the range of 1 to 10 μg/mL. Furthermore, an identical volume of the internal standard solution was added to each calibration standard. Every calibration point contained ISD at ultimate concentration levels of 5 μg/mL. Solutions were used to determine the specific fragmentation pattern for each analyte. The commercially available SQDG, the internal standard, and raw extracts of *Arthrospira* sp. (“Spirulina”) were used. The SQDG 816 and ISD stock solution were diluted with acetonitrile/water (9/1; v/v) with 10 mM ammonium acetate to achieve a concentration of 100 μM SQDG 816/ISD. All solutions were kept at − 20 °C.

### Sample material

Freeze-dried *Spirulina* powder (*Arthrospira* sp.) was obtained from the Institute for Food and Environmental Research (ILU), Nuthetal, Germany. These cyanobacteria were originally cultivated photoautotrophic in a 1500-L tubular photobioreactor PBR 2000 from IGV. Cells were harvested in a modified Zarrouk’s medium under outdoor conditions, i.e., under natural sunlight (with day and night changes), with a pH value of 9.5, and at a temperature over 25 °C. The system was additionally heated and/or artificially illuminated to balance the seasonal temperature fluctuations and overnight respiration losses. Following lyophilization, material was ground to a fine powder. *Spirulina* powder was extracted initially three times with chloroform/methanol (3/2, v/v). After vortexing and ultrasonification for 15 min and centrifuging (5 min, 12,000*g*), the supernatant was removed and collected. The resulting eluate was concentrated to dryness and absorbed into 500 μL methanol. Finally, the mixture was diluted 1:100 (v/v), 1:1000 (v/v), and 1:10,000 (v/v) for further use.

For a screening approach and to compare with contents of the cyanobacteria, commercially available vegetables and a prominent microalgae specie were used. Fresh spinach and basil leaves as well as a *Chlorella* powder (*Chlorella vulgaris*) were purchased from a local organic supermarket in Hamburg, Germany. Spinach and basil leaves were frozen with liquid nitrogen, freeze-dried, and ground with mortar and pestle prior to analysis.

### High-performance thin-layer chromatography

For the chromatographic separation, silica gel 60 high-performance thin-layer chromatography (HPTLC) plates were purchased from Merck KGaA (Darmstadt, Germany). Plates were pre-washed with methanol and activated at 100 °C for 10 min. The samples were applied as bands (8 mm) using an HPTLC autosampler (ATS4, CAMAG AG, Muttenz, Switzerland). Separation was carried out in a twin-trough chamber with chloroform/methanol/acetic acid/water (8.5:1.5:1:0.36, v/v/v/v). Chromatography was consistently performed up to a migration distance of 80 mm. Finally, the remaining solvents were evaporated. Derivatization was performed by dipping the plate 5% copper sulfate in 8% phosphoric acid and afterwards heated for 30 min at 160 °C.

### Sample preparation

The freeze-dried *Spirulina* powder was used for method development. 0.5 g of the powder was then ground in a ball mill for 5 min (frequency 25 Hz, 4 balls *ø* = 1.5 cm; Retsch MM 400, Retsch GmbH, Haan, Germany). Then, 10 mL chloroform/methanol (3/2, v/v) was added and the mixture was ground in a ball mill for 10 min. Ten milliliters chloroform/methanol (3/2, v/v) was added. After vortexing, ultrasonification for 15 min, and centrifuging (5 min, 12,000×*g*), the supernatant was removed and collected in a 10-mL tube. Twenty milliliters chloroform/methanol (3/2, v/v) was added to the residue. After vortexing, ultrasonification for 15 min, and centrifuging (5 min, 12,000×*g*), the supernatant was removed and collected in a 10-mL tube. The last extraction step was repeated three times. The supernatants were combined and evaporated to dryness under nitrogen stream. The residue was re-dissolved in 50 mL chloroform/methanol (3/2, v/v).

### Solid-phase extraction

The sample extract was loaded on a NH_2_-SPE cartridge (6 mL, 500 mg; Macherey-Nagel GmbH & Co. KG, Düren, Germany) that was initially conditioned with 5 mL methanol, 5 mL water, 5 mL 0.1 M HCl, 5 mL water, and 5 mL methanol. Cartridges were washed with 10 mL chloroform/methanol (90/10, v/v) and 10 mL chloroform/methanol (50/50, v/v). SQDGs were eluted with 10 mL chloroform/methanol (80/20, v/v) containing 100 mM ammonium acetate and 2% NH_3_. For removing salts in the eluent, the solution was washed prior to the LC-MS/MS measurement. Therefore, the eluent was mixed with 2 mL 0.9% potassium chloride solution and mixed for 10 min in the ultrasonic bath, then centrifuged for 5 min at 12,000*g*. The organic phase was dried at 40 °C with a vacuum concentrator (RVC 2-25 CDplus, Martin Christ GmbH, Osterode, Germany). Then, the residue was dissolved in 1 mL methanol.

### LC-ESI-MS/MS analysis

The development of the new LC-ESI-MS/MS method as well as the method validation process based on procedures was already published recently [27]. SQDGs were analyzed on an Agilent 1260 Infinity Quarternary LC System (Agilent Technologies Deutschland GmbH, Waldbronn, Germany) coupled to a triple quadrupole API 4000 QTrap mass spectrometer (AB Sciex Germany GmbH, Darmstadt, Germany) equipped with a turbo spray source, operating in negative ion mode, with the following mass spectrometer settings: ion spray voltage: − 4500 V; source gas 1: 16 psi; source gas 2: 0 psi; curtain gas: 10 psi. Separation of analytes was achieved using a Kinetex® C18 reversed-phase column (2.6 μm, 150 mm × 2.1 mm i.d.), equipped with a Kinetex® C18 security guard column (Phenomenex Inc., Torrance, CA, USA), using a constant flow rate of 200 μL/min. Eluent A was water and eluent B acetonitrile/water (9/1; v/v), each with 10 mM ammonium acetate. The elution started with 3% eluent B for 2 min and linearly increased to 99% eluent B within 4 min, which was kept constant for 34 min. Then, the composition was readjusted to 3% eluent B within 2 min, followed by 10 min of re-equilibration.

### Method validation

The LC-MS/MS method was validated in absence of matrices (base calibration). Linearity of the method was determined by analysis of a 10-point calibration curve (*n* = 6). Accuracy and precision of the method were assessed with three different concentrations of quality control samples (QC 1–4). Five replicates of each QC point were analyzed on 1 day to determine the intra-day accuracy and precision. This process was repeated over three different days (*n* = 3) in order to evaluate the inter-day accuracy and precision. The recovery rate was calculated as follows: [RE = (*c*_sample_ − *c*_endogen_) × 100/*c*_spiked_].

The limit of detection (LOD) was determined by the calibration method. A calibration curve (*n* = 4) in the range of the presumed detection limit was established and measured (*n* = 3). The parameters of the resulting regression line were then used to determine LOD and limit of quantitation (LOQ).

## Results and discussion

### Method development

Previously published methods for determining sulfolipids indicated limitations in their selectivity or sensitivity. Most of these methods do not differentiate between single SQDGs or SQMGs, but determine them as sum parameters. In the present study, a method development for selective and sensitive SQDG determination is described. For a sensitive and selective detection of the SQDGs using mass spectrometry, multiple reaction monitoring (MRM) was applied. Besides sensitive and selective detection of the SQDGs, another advantage using MRM method was the achievement of higher sensitivity and better repression of interferences of the complex backgrounds. MRM transitions of the different SQDGs were obtained by direct flow injection of a crude *Arthrospira* sp. extract (acetonitrile/water, 9/1, v/v) with 10 mM ammonium acetate into the ESI source in positive and negative ionization modes. The raw extract was used, because there was only one commercially available SQDG standard and the scope was to develop a method for several SQDGs. For each precursor ion being selected based on the full scan (*m/z* 50–1000), the three most intense fragment ions (product ions) were determined.

**Fig. 1:**
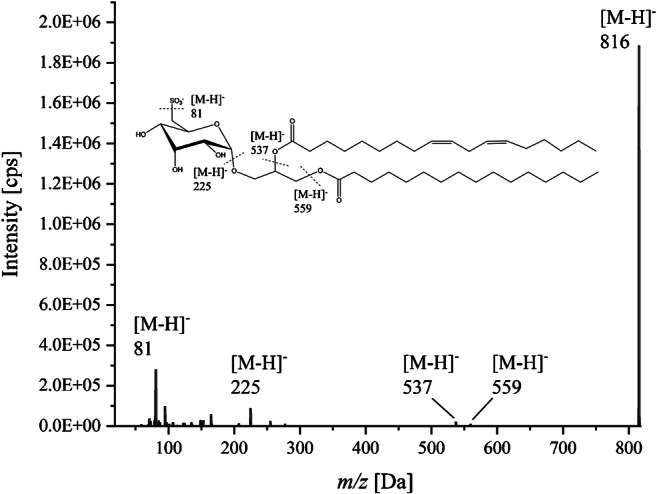
ESI-MS-spectrum of the SQDG 816 [M-H] ¯ and the possible fragmentation pattern

Table 1 summarizes the precursor and fragment ions of all compounds tested in the present study. Mass analyzer settings were optimized for all analytes to maximize the transmission and sensitivity of each characteristic mass transition. These optimizations were acquired automatically by using autotune mode provided by the Analyst® software 1.6.1 (AB Sciex Germany GmbH, Darmstadt, Germany). Attention was paid to the fragmentation pattern and only mass numbers with fragments typical for SQDGs were included. Such fragments of SQDGs are *m/z* 81, *m/z* 125, *m/z* 153, *m/z* 165, *m/z* 225, and *m/z* 255. A comprehensive overview of the fragmentation pattern of selected SQDGs was published by Zhang et al. [23]. Exemplarily, Fig. 1 shows the fragmentation of the SQDG 816. The nomenclature of the various SQDGs used in this work is based on the mass [M-H] ¯. This means, for example, SQDG 817 has the mass *m/z* 816 [M-H] ¯ as main fragment in the MRM method. As obvious from Fig. 1, the typical fragments *m*/*z* 81 and *m*/*z* 225 were detected as full scan in negative mode. Additionally, the fragments *m*/*z* 537 and *m*/*z* 559 represent the masses of the loss of one fatty acid. With the help of these fragments, a conclusion about the bound fatty acids can be made. The fragment *m*/*z* 537 corresponds with the loss of the fatty acid 18:2 (linoleic acid) and *m*/*z* 559 with the fatty acid 16:0 (palmitic acid). A further identification of the fatty acids was not always possible, because some sulfolipids are isobaric compounds with combinations of fatty acids equal to each other, but different in their position. In the negative mode only, the fragment of the intact fatty acid loss was detected.

**Table 1:** MRM transitions and MS parameters in negative ion mode for all SQDGs and the internal standard (ISD)

Analyte	Rt (min)	M (g/mol)	MRM transition (*m/z*)	DT (ms)	DP (V)	EP (V)	CE (P)	CXP (V)
792 (ISD)	20.5	794	792.0 → 96.0	150	− 155	− 10	− 128	− 15
			792.0 → 58.0	150	− 155	− 10	− 130	− 1
			792.0 → 80.0	150	− 155	− 10	− 128	− 3
555	9.23	555	555.0 → 80.0	150	− 130	− 10	− 100	− 3
			555.0 → 94.0	150	− 130	− 10	− 80	− 5
			555.0 → 224.0	150	− 130	− 10	− 64	− 15
765	19.7	765	765.0 → 80.0	150	− 110	− 10	− 120	− 11
			765.0 → 224.0	150	− 110	− 10	− 64	− 15
			765.0 → 94.0	150	− 110	− 10	− 112	− 5
787	15.4	787	787.3 → 80.0	150	− 155	− 10	− 112	− 11
			787.3 → 224.0	150	− 155	− 10	− 64	− 1
			787.3 → 164.0	150	− 155	− 10	− 11	− 11
789	17.4	789	789.2 → 80.8	150	− 165	− 10	− 130	− 1
			789.2 → 224.7	150	− 165	− 10	− 64	− 17
			789.2 → 164.7	150	− 165	− 10	− 14	− 11
791	11.7	791	791.0 → 81.0	150	− 160	− 10	− 104	− 3
			791.0 → 225.0	150	− 160	− 10	− 64	− 5
			791.0 → 165.0	150	− 160	− 10	− 80	− 1
793	25.7	793	793.0 → 80.8	150	− 135	− 10	− 130	− 9
			793.0 → 224.8	150	− 135	− 10	− 17	− 17
			793.0 → 79.9	150	− 135	− 10	− 1	− 1
801	16.6	801	801.3 → 81	150	− 150	− 10	− 130	− 11
			801.3 → 152.9	150	− 150	− 10	− 70	− 9
			801.3 → 224.9	150	− 150	− 10	− 62	− 15
803	18.9	803	803.4 → 80.6	150	− 185	− 10	− 128	− 3
			803.4 → 224.8	150	− 185	− 10	− 64	− 17
			803.4 → 164.5	150	− 185	− 10	− 80	− 11
805	23.1	805	805.0 → 81.0	150	− 65	− 10	− 114	− 3
			805.0 → 80.0	150	− 65	− 10	− 126	− 1
			805.0 → 224.0	150	− 65	− 10	− 68	− 17
807	29.7	807	807.2 → 80.6	150	− 130	− 10	− 126	− 1
			807.2 → 224.9	150	− 130	− 10	− 66	− 11
			807.2 → 94.8	150	− 130	− 10	− 108	− 3
813	15.7	813	813.3 → 80.9	150	− 160	− 10	− 130	− 1
			813.3 → 152.7	150	− 160	− 10	− 72	− 13
			813.3 → 94.7	150	− 160	− 10	− 126	− 1
815	18.4	815	815.4 → 80.9	150	− 205	− 10	− 118	− 3
			815.4 → 152.7	150	− 205	− 10	− 64	− 15
			815.4 → 94.7	150	− 205	− 10	− 106	− 5
817	21.3	817	817.0 → 81.0	150	− 230	− 10	− 120	− 11
			817.0 → 225.0	150	− 230	− 10	− 66	− 17
			817.0 → 165.0	150	− 230	− 10	− 72	− 9
819	26.1	819	819.3 → 80.8	150	− 150	− 10	− 116	− 1
			819.3 → 225.1	150	− 150	− 10	− 68	− 3
			819.3 → 164.7	150	− 150	− 10	− 72	− 13
821	36.0	821	821.5 → 80.8	150	− 140	− 10	− 130	− 1
			821.5 → 225.1	150	− 140	− 10	− 72	− 1
			821.5 → 95.0	150	− 140	− 10	− 120	− 5
837	14.6	837	837.2 → 80.9	150	− 80	− 10	− 124	− 3
			837.2 → 224.7	150	− 80	− 10	− 66	− 15
			837.2 → 165.1	150	− 80	− 10	− 74	− 1
839	16.3	839	839.3 → 80.0	150	− 110	− 10	− 120	− 5
			839.3 → 224.0	150	− 110	− 10	− 62	− 17
			839.3 → 164.0	150	− 110	− 10	− 76	− 11
841	18.6	841	841.3 → 80.9	150	− 200	− 10	− 124	− 11
			841.3 → 224.7	150	− 200	− 10	− 72	− 15
			841.3 → 164.7	150	− 200	− 10	− 82	− 9
843	22.7	843	843.3 → 80.9	150	− 170	− 10	− 118	− 3
			843.3 → 224.9	150	− 170	− 10	− 68	− 17
			843.3 → 94.7	150	− 170	− 10	− 110	− 3
845	28.0	845	845.4 → 80.9	150	− 175	− 10	− 118	− 3
			845.4 → 224.6	150	− 175	− 10	− 70	− 11
			845.4 → 94.8	150	− 175	− 10	− 120	− 15
847	10.4	847	847.3 → 81.1	150	− 170	− 10	− 122	− 11
			847.3 → 224.7	150	− 170	− 10	− 70	− 15
			847.3 → 95	150	− 170	− 10	− 110	− 1
833	13.0	833	833.5 → 81.1	150	− 100	− 10	− 124	− 3
			833.5 → 224.8	150	− 100	− 10	− 66	− 19
			833.5 → 165	150	− 100	− 10	− 75	− 11
849	11.9	849	849.4 → 80.8	150	− 160	− 10	− 130	− 11
			849.4 → 224.7	150	− 160	− 10	− 72	− 3
			849.4 → 94.6	150	− 160	− 10	− 130	− 3
855	11.0	855	855.0 → 80.0	150	− 145	− 10	− 128	− 1
			855.0 → 224.0	150	− 145	− 10	− 74	− 11
			855.0 → 164.0	150	− 145	− 10	− 80	− 9
867		867	867.0 → 80.0	150	− 175	− 10	− 128	− 3
			867.0 → 224.0	150	− 175	− 10	− 78	− 15
			867.0 → 164.0	150	− 175	− 10	− 86	− 1
871		871	871.0 → 80.0	150	− 195	− 10	− 126	− 3
			871.0 → 224.0	150	− 195	− 10	− 72	− 17
			871.0 → 164.0	150	− 195	− 10	− 80	− 7

*Qnt*, quantifier; *Qal 1*, qualifier 1; *Qal 2*, qualifier 2; *DT*, dwell time; *DP*, declustering potential; *EP*, entrance potential; *CE*, collision energy; *CXP*, collision cell exit potential

The measurement was carried out in negative mode, because sulfolipids are easily deprotonated to form the quasi-molecular ion [M-H] ¯, due to their strongly acidic sulfate group. The negative and the positive ion modes were used during this study, but the results of the ionization in the negative mode were much more meaningful. The negative ion mode is particularly suitable for electrophilic substituents such as the sulfonic acid group, which was detected a hundred times more sensitive compared to the positive ion mode. The intensity in the positive mode was so low that the software was not able to record the data as an MRM approach. It is possible that the formation of the positive adducts was not stable enough so that no suitable fragments could be recorded, as the formation of the sulfonic acid ion with the cleavage of a proton yielded more stable fragments. This was already observed by Keusgen et al. [28]. Xu et al. [29] observed the same phenomenon for SQDGs when using positive ionization mode. Although they tried to use different modifiers for stabilizing the SQDG fragments in positive mode, they did not reach satisfactorily results [29]. Lu et al. [30] published a study about glycolipids. They showed that the positive mode is suitable for neutral galactolipids. Monogalactosyl diacylglycerol (MGDG) and digalactosyl diacylglycerole (DGDG) are preferentially detected in positive mode, but SQDGs and phosphoglyceroles (PG) as negative charged glycolipids are more efficiently ionized in negative mode [30]. More recent publications did not mention the positive mode anymore. For this reason, negative ion mode was used for all further measurements and automatic compound optimization was performed in the present study.

In order to clearly distinguish SQDGs from other substances in the same mass range, three analytes with similar mass and similar properties were selected. When using dipalmitoylphophatidylcholine (734 g/mol) and soya lecithin (644 g/mol), no peaks in the target range of *m/z* 50–1000 could be found in negative ionization mode in the full scan. For ceramide (780 g/mol), the fragments *m/z* 601 and *m/z* 628 were detected in negative ionization mode. However, their fragmentation patterns did not show typical fragments of a SQDG. Therefore, it was assumed that the analytes found were indeed sulfolipids and that they could be distinguished from molecules of the same class with similar properties by using MRM.

In the *Spirulina* powder extract, 25 SQDGs and one SQMG were detected. The fragment pattern was created using Analyst® software 1.6.1 (AB Sciex Germany GmbH, Darmstadt, Germany) and the fragmentations were used for the detection following the liquid chromatographic separation. Fragments of the individual SQDGs hardly differed, but, because of the second dimension added by liquid chromatography, this similarity was not significant anymore. The conditions for the liquid chromatographic system were selected based on a literature survey and an empirical approach. According to Keusgen et al. [28], a RP18 column and a flow of 200 μL/min were used. SQDGs have a large apolar part in their chemical structure, resulting from the fatty acids. Different SQDGs can only be distinguished by resolving the different fatty acids. Due to the apolar properties of the separation column, the SQDGs can be retarded for different lengths of time due to van der Waals forces, thus being separated from each other. According to Robbins et al. [31], 10 mM ammonium acetate in water and 10 mM ammonium acetate in acetonitrile/water (9/1, v/v) were used as mobile phases. The addition of ammonium acetate as a buffer was used so that the SQDGs were largely negatively charged and can thus be better ionized in the negative ion mode. Robbins et al. [31] showed a similar ionization behavior of sulfides. Consequently, this additive was chosen for the SQDG method, because the ionization is highly depending on the sulfate group [31].

**Fig. 2:**
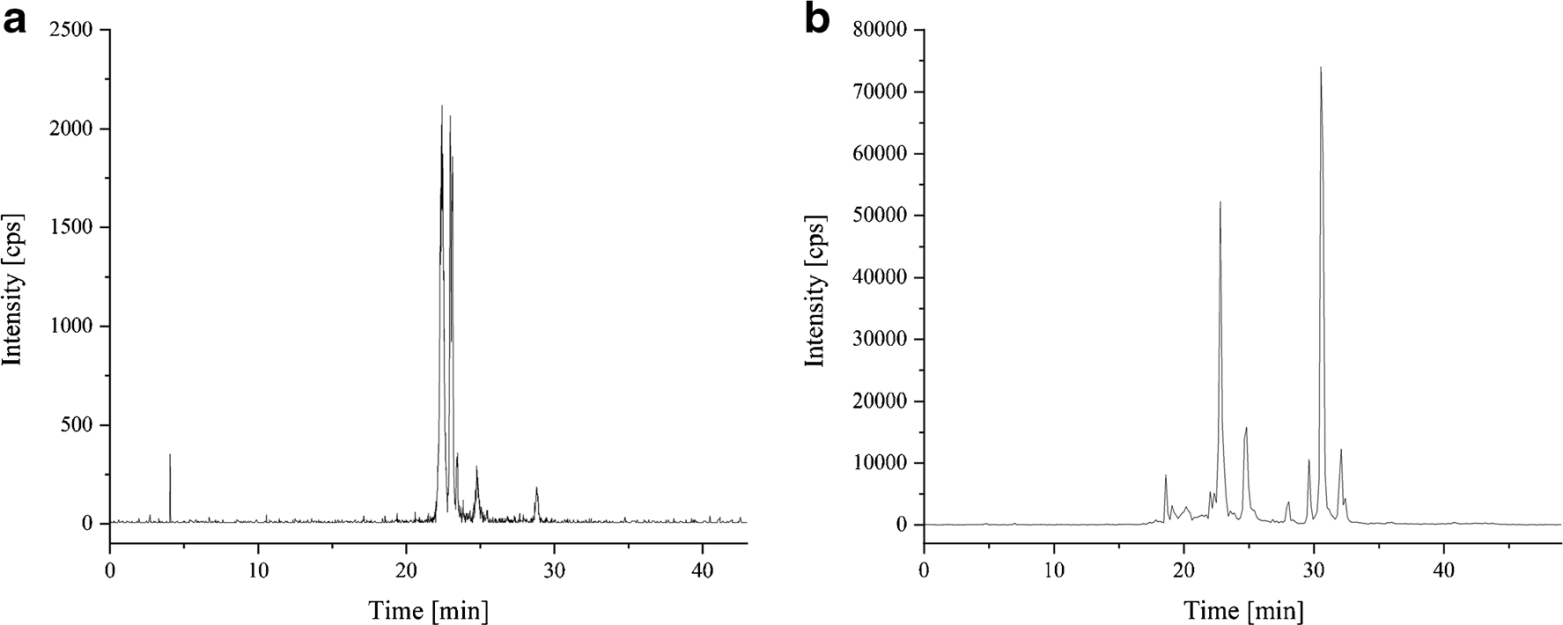
Total ion chromatogram (TIC) depending on the separation gradient used. **a** Original gradient and **b** final gradient

Figure **2** shows the total ion chromatogram of the *Arthrospira* sp. extract (1:100 diluted with acetonitrile) separated with the first HPLC gradient (Fig. 2a). In comparison, Fig. 2b shows the TIC of the MRM *Arthrospira* sp. extract (1:100 diluted with acetonitrile), which was measured with the final HPLC gradient. It is noticeable that when using the first gradient, the peaks overlap in a narrow range between the 22nd and 24th minutes and no separation of the analytes took place. In contrast, the chromatogram of the final gradient shows that the peaks extend over a range of 27 min (9th–36rd minutes) and an almost baseline separation between the peaks can be seen.

### Sample preparation

**Fig. 3:**
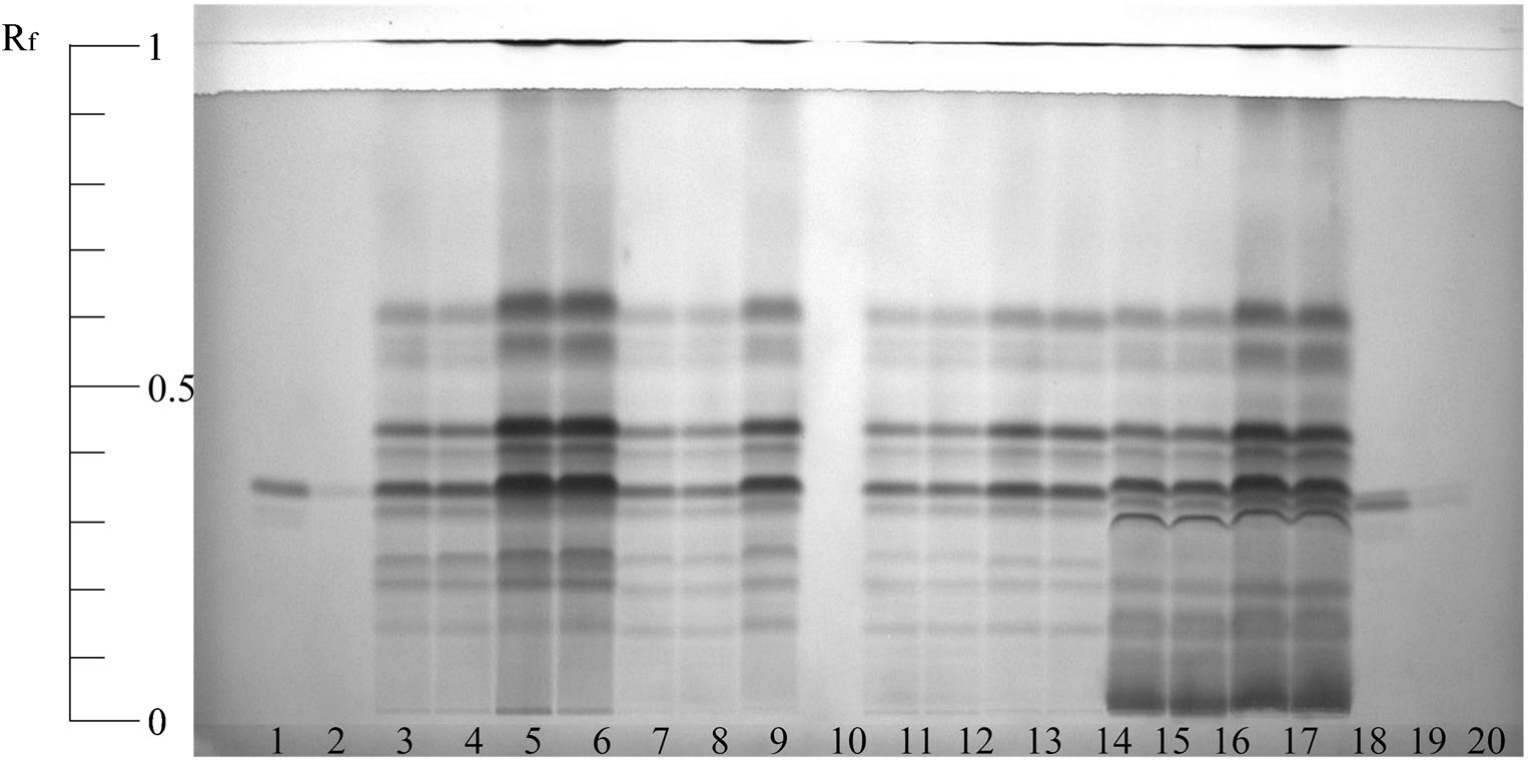
HPTLC of lipid extracts from *Arthrospira* sp. extracted with different solvents (according to Table 2). Derivatization was performed with 5% copper sulfate in 8% phosphoric acid and then heated for 30 min at 160 °C

For the development of a quantitative extraction method, freeze-dried and powdered *Arthrospira* sp. was used. As freeze-dried *Spirulina* powder or plant material is complex matrices, a comprehensive sample preparation is essential. With regard to the SQDG analysis, it is important that chlorophylls and other lipids are removed as completely as possible to not interfere chromatographic separation. The individual steps (cell disruption, extraction solvent, SPE as clean-up procedure) of the sample preparation were characterized for their effectiveness using HPTLC. Initially, various extraction agents were tested for their solubility of SQDGs. A process for the extraction of lipids, including sulfolipids, which is often described in the literature, is the so-called Folch extraction, which is applied to animal tissues as well as plant materials [32]. The process is based on the extraction of a mixture of chloroform and methanol in a ratio of 3:2 by ultrasonification and agitation. However, as chloroform in particular is highly affine for chlorophyll, further extraction agents were tested to reduce the amount of chlorophyll and to determine whether other combinations of solvents achieve a higher partition coefficient with regard to the sulfolipids. Figure 3 shows the lipids detected in the crude extracts after applying different solvent systems. The samples were then analyzed by LC-MS/MS for a more precise assessment of the concentration. The peak areas of the most intense representative SQDG (*m/z* 815) were considered for comparing (Fig. 3, Table 2).

**Table 2:** List of solvent mixtures tested for the extraction of SQDGs from *Arthrospira* sp.

Position^1^	Solvent mixture	Weight of *Arthrospira* sp. (g)	Analyte peak area (counts, SQDG *m/z* 815)
1, 19	SQDG standard		
2, 20	SQDG standard		
3, 4	Chloroform/methanol (3:2)	0.5	4.02E+06
5, 6	Chloroform/methanol (3:2)	1	6.34E+06
7, 8	Methyl tert-butyl ether (MTBE)/methanol (3:2)	0.5	2.27E+06
9, 10	MTBE/methanol (3:2)	1	4.67E+06
11, 12	Ethanol	0.5	2.61E+06
13, 14	Ethanol	1	3.56E+06
15, 16	Ethanol/methanol/water (3:3:1.5)	0.5	3.32E+06
17, 18	Ethanol/methanol/water (3:3:1.5)	1	5.37E+06

^1^Position in the gel presented in Fig. 3

The SQDG standard (Fig. 3, lanes 1, 2, 19, and 20) showed a strong band with a retention factor of 0.32. When comparing this band to the one of the SQDG standard, all extraction agents showed a strong band at the corresponding height, indicating that all the extraction agents investigated provide a strong affinity towards SQDGs. However, it also showed that other unspecific substances were extracted in addition to the SQDGs. This is especially valid for the extraction approach with ethanol/methanol/water (3:3:1.5) (Fig. 3, lanes 15–18). Here, considerably more unspecific and partly more intensive bands of contaminants were present. The different solvent compositions showed no significant influence on the number and diversity of the extracted sulfolipids. There were no differences between them when using the individual extraction agents. Cutignano et al. [25] used a system consisting of MTBE and methanol to extract complex lipids from marine microalgae. However, as the extraction of other lipid classes was similar to chloroform/methanol, this solvent system was not pursued any further. In summary, the composition of chloroform and methanol appeared to be suitable. Compared to the other extraction agents, this mix showed the highest extraction rate with similar or low contamination of non-target substances under the same treatment. Chloroform and methanol in different compositions are still the traditional solvent systems, which are used to extract lipids from different tissues [32, 33]. More actual methods often rely on these older extraction methods [24, 26]. Therefore, the result that chloroform/methanol is (still) the best system to extract different lipid classes in high amounts is not surprising.

**Fig. 4:**
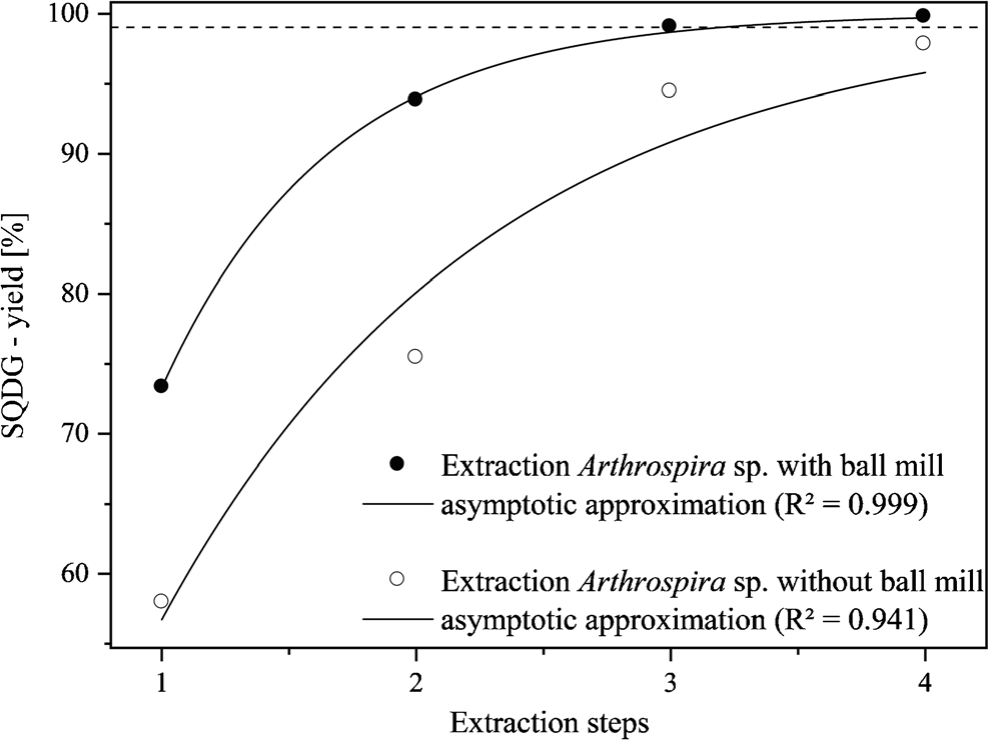
Extraction efficiency with regard to SQDG extraction depending on ball mill application

The next step within the development of the extraction process was to establish a quantitative isolation of SQDGs. Generally, sulfolipids are located in the thylakoid membranes of plant cells [34, 35]. Their content varies between 4 and 7% of total leaf lipids [36]. In marine plants, cyanobacteria, and microalgae, the content can be up to 66% of total polar lipids [37]. As SQDGs are firmly embedded in the membranes, a cell disruption is required to be able to isolate them quantitatively. Ball mills are often used to break down cell walls of plants or bacteria, and can be used to study substances that are located inside the cell in the specific organelles [38–40]. It is a mechanochemical approach to break the cell walls and extract substances with chemical solvents at the same time. Figure 4 shows the extraction steps with and without cell disruption using a ball mill prior to the extraction. Using a ball mill leads to extraction rates over 99%, when applying three extraction steps, and without using a ball mill, only 94% were extracted after three extraction cycles. The dotted line shows the extraction rate of 99%.

### Development of a solid-phase extraction clean-up procedure

As obvious on the HPTLC plate (Fig. 3), SQDG extracts still show numerous impurities due to a variety of other lipophilic substances. This can cause falsified results in the follow-up quantification by co-elution of these substances in the LC-MS/MS method. In order to avoid this, a SPE was carried out for further purification of the extract. Due to their amphiphilic character, the sulfolipids show potential for the enrichment on different solid phases. Consequently, two different selectivities were tested: on the one hand, a C18-modified silica gel with affinity to the non-polar tail group of the SQDGs, and on the other hand, two phases with anion exchange functionality (DEAE-modified Sephadex® and aminopropylated silica gel). The latter solid phases are selective for the sulfate group, which is completely dissociated under neutral pH conditions. To assess the effectiveness of the solid phases used, the individual eluates of the application, washing, and elution were analyzed individually by HPTLC to detect occurrence of unspecific substances. Their removal due to the SPE was determined on the basis of the internal standard. The standard was added to the raw extract prior to the SPE. The recovery rate of the internal standard was 41% for C18-SPE columns, 76% for DEAE-Sephadex^®^, and 73–96% for amino-propylated silica gel. When using DEAE-Sephadex®, the best separation of the matrix was achieved, but the handling of the separation material was very complex. The separation on amino-propylated silica gel also provided very good results and was easier to handle and therefore used in the final application.

### Method validation

Initially the quantification of the SQDGs should be performed with an external calibration curve only. Therefore, a commercial standard (SQDG 816, Avanti Polar Lipids Inc., Alabaster, AL, USA) was diluted and 12 calibration points from 0.001 to 500 μM solutions were analyzed (*n* = 7).

**Fig. 5:**
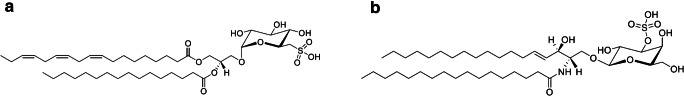
Structures of SQDG (**a**) and cerebroside (**b**)

However, the results were not distributed normally. Due to the lack of normally distributed results, an internal standard was added to the calibration solutions. Therefore, a cerebroside with the molar mass of 794 g/mol was chosen. Cerebrosides show chemical and structural similarities to the SQDGs and are generally not found in plant tissues, but mainly in the nerve tissue of animals [41]. Consequently, it was selected as the internal standard for the quantification of sulfolipids from plant matrices. Figure 5 shows the structures of a typical SQDG (Fig. 5a) and the cerebroside (Fig. 5b), which was used as internal standard. It can be seen that the substances are similar in some structural features like the sulfonated hexose derivate, but the fatty acid esterified can differ and lead to isobaric compounds.

All samples were freeze-dried powders. The differences between the matrices can be found especially in the kind of cell membranes. They differ from plant cell membranes to gram-positive membranes in cyanobacteria. The outer layer is particularly different. In the present study, samples were treated with conditions around 1 kPa and temperatures of less than − 40 °C during the freeze-drying process, leading to more fractal cell walls, due to the formation of ice crystals [42]. Therefore, the differences in the sample matrices became less important. Another reason for the little influences of the sample matrices was that the main concentration of SQDGs can be found in the thylakoid membranes of the chloroplast, which should be similar in all samples. The small impact of the matrix differences can be seen in the similar recovery rates for the different matrices.

The same concentration of internal standard was added to the external calibration standards in order to calculate different fragmentation behaviors between each analysis run. The data of the calibration curves acquired in this way was then normally distributed.

**Fig. 6:**
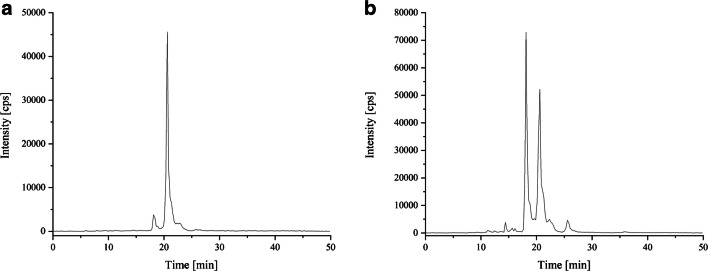
Chromatograms of the calibration measurements. **a** SQDG standard 1 ppm, retention time 19.5, and ISD 5 ppm, retention time 20.5 min; **b** SQDG standard 10 ppm, retention time 19.5, and ISD 5 ppm, retention time 20.5 min

Figure 6 shows exemplarily the lowest and the highest calibration standards consisting of SQDG 816 (Fig. 6a) and the cerebroside, which was used as internal standard (Fig. 6b).

The analysis of SQDGs and one SQMG in freeze-dried cyanobacteria and plant material was subsequently validated in terms of linearity, precision, and accuracy on intra- and inter-day, recovery, and lower limit of quantification (LLOQ) according to well-accepted guidelines [43]. The determination coefficients (*R*^2^) of the calibration curves showed an excellent correlation with a high mean value (0.989 ± 0.008) (Table 3). According to the guidelines of the FDA, the coefficient of variation (CV) shall be < 15%. Methods with a CV < 15% can be considered precise [43]. The calculated CV for the external and internal standards (*n* = 6) ranged between 4.4 and 14.67%, indicating that the method meets the FDA’s requirements in terms of precision. The LOQ determined for quantitation was 1.44 μg/mL. The recovery depended on the matrix, but achieved good recovery rates for representative analytes (*Arthrospira* sp. 83%, *C. vulgaris* 95.8%, basil 89.5%, spinach 88%). So far there is only one publication on a semi-quantitative determination of single SQDGs in *Arthrospira* sp., described by Antonelli et al. [24]. They achieved a comparatively better LOQ (0.41 μg/mL), but almost similar recovery rates (89–110%), and presented a similar range for linearity of the external calibration (0.75–12.5 μg/mL).

**Table 3:** Curve parameters for the calibration of the LC-ESI-MS/MS SQDG quantification method

Analyte	LOQ (μM)	Slope (mean ± SD)	*R*^2^ (mean ± SD)	Intercept (mean ± SD)
SQDG	1.44 μg/mL	0.330 ± 0.021	0.989 ± 0.008	0.018 ± 0.009

*LLOQ*, limit of quantification; *R*^*2*^, relative standard deviation; *Intercept*, standard deviation of the procedure; *CV*, coefficient of variation

### Method application

Following the successful validation of the quantification method, the application to real samples followed. For this purpose, different plant matrices were selected. This was to test the application of the method on different freeze-dried and powdered plant matrices. Spinach was selected, because it was the first plant to demonstrate the presence of sulfolipids and other comprehensive studies have already been published [6, 44]. There are no published data for basil yet. *C. vulgaris* was chosen as the most prominent representative of microalgae, because of its importance as a dietary supplement in Europe. The SQDG content of selected microalgae has been published to some extent. These microalgae showed a large difference in their SQDG content (*Heterosigma carterae*, 5.3 mg/g [28]; *Dictyochloris fragrans*, 0.9 mg/g [42]). As already described, there are some methods that quantify SQDGs as sum parameters. Methods that can be used to distinguish between the quantification of individual SQDGs are only published for *Arthrospira* sp. by Antonelli et al. [24].

**Fig. 7:**
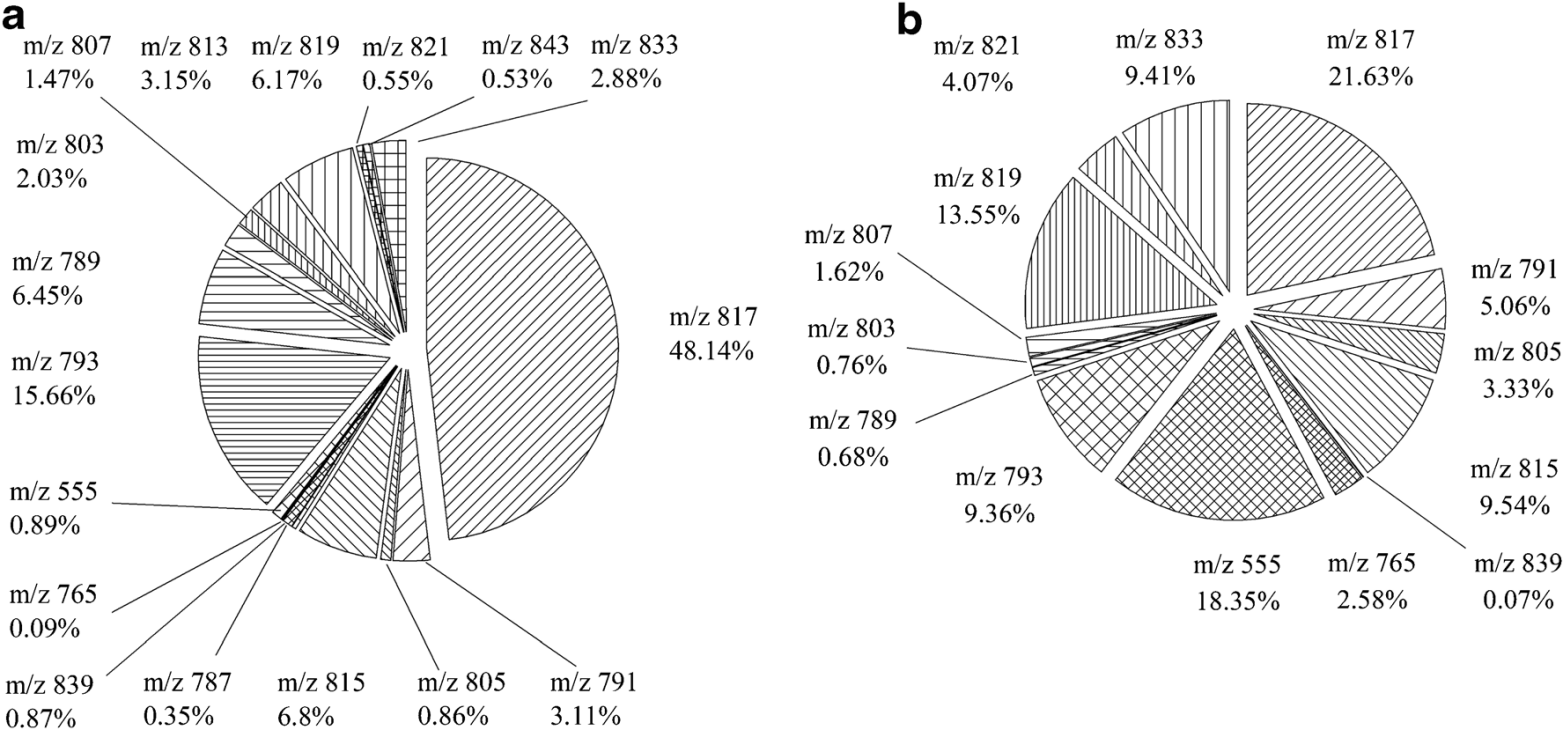
Shares of the different SQDG masses of the total distribution in *C. vulgaris* (**a**) and *Arthrospira* sp. (**b**)

**Fig. 8:**
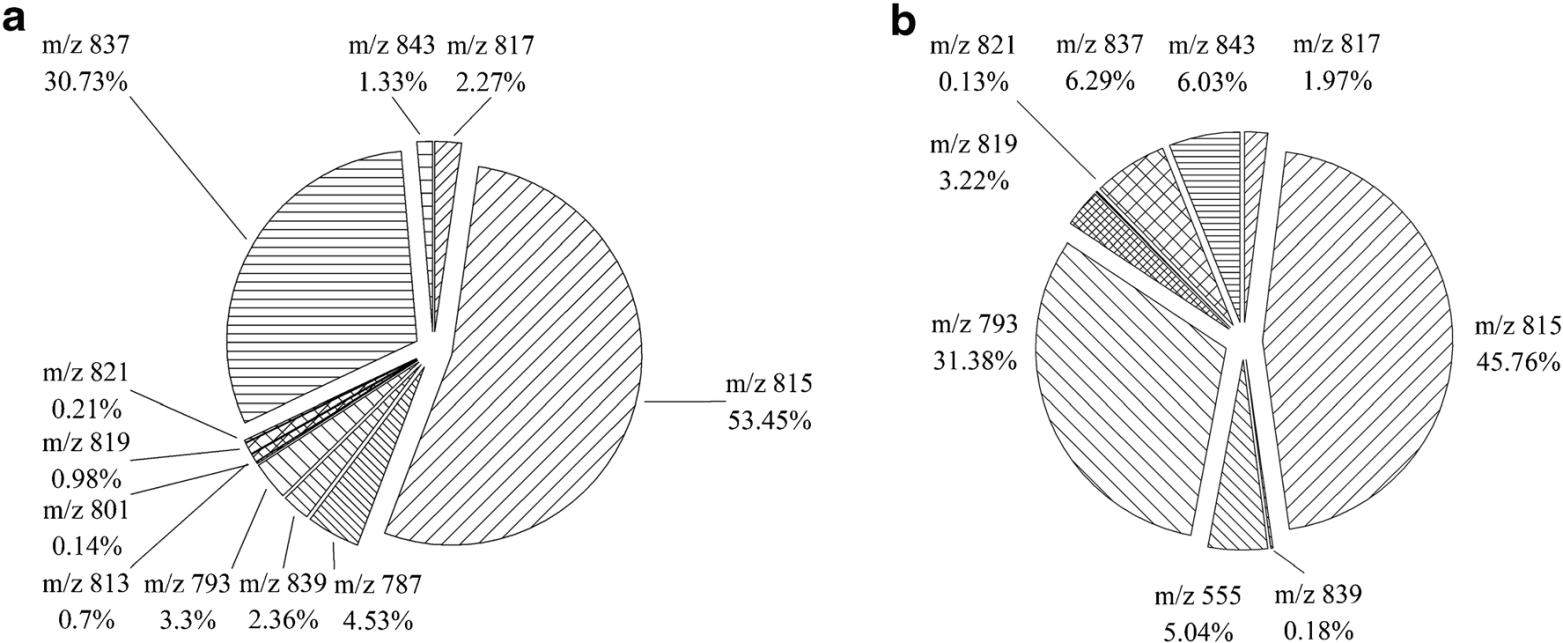
Shares of the different SQDG masses of the total distribution in spinach leaves (**a**) and basil leaves (**b**)

In the present study, the total SQDG content varied from 1.7 mg/g (*Arthrospira* sp.) to 1 mg/g (*C. vulgaris*) and 702 mg/g (spinach) and 387 mg/g (basil), respectively. The SQDG content previously reported for spinach was 824 μg/g dry weight [44]. This content is comparable to the content found using this newly described method. There are no data about the composition of SQDGs in spinach, basil, and *C. vulgaris*, so far. Table 1 shows the SQDG masses measured with the developed method. Figures 7 and 8 show the SQDG masses found with the described method.

When looking at Figs. 7 and 8, it is noticeable at a first glance that SQDG 815 is represented in the two cormophytes as the dominant SQDG with proportions between 45 and 53%. In contrast to the *Chlorella* and the *Spirulina* samples with SQDG 817 being the most frequent SQDG, *m/z* 815 is a C18 fatty acid with a double bond. In addition, cormophytes tend to be more prone to SQDGs with masses higher than 800 g/mol, while the masses of SGDQs in *Spirulina* and *Chlorella* varied between 700 and 800 g/mol. As a result, *Spirulina* and *Chlorella* samples showed a higher diversity. On average, about 15 different SQDGs were identified in those samples as compared to nine SQDGs in basil and ten SQDGs in spinach. In summary, when marine organisms are compared with cormophytes, a correlation between the decrease in *m/z* 815 and a related increase in diversity can be observed. Antonelli et al. [24] described nine main SQDGs in *Athrospira* sp. Table 4 shows a comparison of the content of SQDGs described by Antonelli et al. [24] and the yielded content of the same SQDG with the newly described method. Only the SQDGs described in both studies are shown [24].

**Table 4:** SQDG content in *Spirulina* sp. according to different methods

SQDG	SQDG content^1^ (%)	SQDG content^2^ (%)
555	49.45	18.35
791	3.63	5.06
793	5.89	9.36
815	4.94	9.54
817	12.93	21.63
819	2.55	13.55

^1^According to Antonelli et al. [24]; ^2^according to the method presented herein

However, a comparison between both studies is difficult for several reasons. Obviously, differences in the contents found might be due to different cultivation conditions, processing, and storage of *Spirulina* sp., sample materials are the same for both methods. However, there are also major differences in the sample preparation methods: Here, the main difference is the purification of the raw extracts. The different SPE phases can possibly lead to shifted SQDG patterns, depending on the phases used, which should be ideally be checked by the analysis of the recovery of standard compounds. Another important difference can be found in the mass spectrometric method applied. While Antonelli et al. [24] were able to differentiate regioselectivity between SQDGs with the same fatty acids in sn1 and sn2 positions by using UHPLC-HRMS, the present method focused even more on a precise quantification using MRM.

## Conclusions

The development of a reliable and quantitative method for the isolation of SQDGs from cyanobacteria and selected leafy vegetables (spinach, basil), a clean-up with SPE, and a sensitive LC-ESI-MS/MS method for a simultaneous identification and quantitation of different, intact SQMGs and SQDGs was introduced. The precision and accuracy of this method for analyzing freeze-dried cyanobacteria, microalgae, and plant material have been successfully demonstrated exemplarily at hand of these different matrices.

The sample preparation is a slightly more complex as compared to previous methods, but the result is nevertheless convincing and the method developed can now be used to study biochemical mechanisms of the formation of SQDG under certain growth conditions. Further, the importance of SQDG and SQMG in raw materials used for food processing and consequences for human nutrition can be characterized. However, the present study cannot yet reveal the reason for the high SQDG diversity, but with the developed quantification method, it provides a useful tool to answer this and further questions.
